# Characterization of interactions between inclusion membrane proteins from *Chlamydia trachomatis*

**DOI:** 10.3389/fcimb.2015.00013

**Published:** 2015-02-11

**Authors:** Emilie Gauliard, Scot P. Ouellette, Kelsey J. Rueden, Daniel Ladant

**Affiliations:** ^1^Unité de Biochimie des Interactions Macromoléculaires, Département de Biologie Structurale et Chimie, Institut Pasteur, Centre National de la Recherche Scientifique, Unité Mixte de Recherche 3528Paris, France; ^2^Université Paris Diderot, Sorbonne Paris Cité, Cellule PasteurParis, France; ^3^Division of Basic Biomedical Sciences, Sanford School of Medicine, University of South DakotaVermillion, SD, USA

**Keywords:** *Chlamydia*, inclusion membrane proteins, Inc protein, bacterial two-hybrid system, BACTH, protein-protein interactions

## Abstract

Chlamydiae are obligate intracellular pathogens of eukaryotes. The bacteria grow in an intracellular vesicle called an inclusion, the membrane of which is heavily modified by chlamydial proteins called Incs (Inclusion membrane proteins). Incs represent 7–10% of the genomes of *Chlamydia* and, given their localization at the interface between the host and the pathogen, likely play a key role in the development and pathogenesis of the bacterium. However, their functions remain largely unknown. Here, we characterized the interaction properties between various Inc proteins of *C. trachomatis*, using a bacterial two-hybrid (BACTH) method suitable for detecting interactions between integral membrane proteins. To validate this approach, we first examined the oligomerization properties of the well-characterized IncA protein and showed that both the cytoplasmic domain and the transmembrane region independently contribute to IncA oligomerization. We then analyzed a set of Inc proteins and identified novel interactions between these components. Two small Incs, IncF, and Ct222, were found here to interact with many other Inc proteins and may thus represent interaction nodes within the inclusion membrane. Our data suggest that the Inc proteins may assemble in the membrane of the inclusion to form specific multi-molecular complexes in an hierarchical and temporal manner. These studies will help to better define the putative functions of the Inc proteins in the infectious process of *Chlamydia*.

## Introduction

The *Chlamydiae* are obligate intracellular bacterial pathogens of humans and animals, causing acute and chronic diseases (Schachter et al., 1973; Brunham et al., 1985; Grayston, 1992; Taylor-Robinson et al., 1992; Mabey et al., 2003). *Chlamydia trachomatis* is a human pathogen responsible for trachoma and the most common sexually transmitted bacterial infection in the world (about 2.8 million infections are reported each year in the United States alone). In most cases, a *Chlamydia trachomatis* infection is asymptomatic in women. However, the infection damages the reproductive organs and, in the most severe cases, can cause infertility (Stamm, 1999).

The Chlamydiales differ from other intracellular pathogens by their biphasic developmental cycle (AbdelRahman and Belland, 2005 for review). They exist in two forms: the elementary body (or EB) and the reticulate body (or RB). *Chlamydia* invades a cell as an infectious EB form, and, inside the host cell, differentiates into the non-infectious RB form to multiply by binary fission. Unspecified signals lead to the re-differentiation of RBs back to EBs, which results in release of infectious Ebs in the external medium. Importantly, *Chlamydia* grows within a host cell-derived vesicle, in the membrane of which it inserts specific bacterial proteins, called Inc proteins, to generate a so-called “inclusion.” These Inc proteins are thus hypothesized to facilitate communication between *Chlamydia* and the host cell.

The Inc proteins have two major characteristics: an N-terminal type III secretion signal that is necessary for their secretion out of the bacterium and a hydrophobic region consisting of at least two transmembrane helices that allows insertion into the inclusion membrane. Generally, both the N- and C-terminal regions of the Inc are exposed to the host cell cytosol. Based on these characteristics, Dehoux et al. (2011), through a bioinformatics analysis of chlamydial genomes, identified 59 putative Incs in *Chlamydia trachomatis* and 107 in *Chlamydia pneumoniae*. The *inc* genes thus represent 7–10% of the genomes of *Chlamydia*, which is remarkable given that these species have undergone a massive reductive evolution of their genome (~1 Mbp). The presence of so many Inc proteins suggests that they play a key role in the development and virulence of *Chlamydia*. However, their functions remain largely unknown, and, as genetic tools have only recently been implemented for this bacterium (Wang et al., 2011), functional analysis of these components is still very limited.

One function of Inc proteins may be to interact with host cell proteins to facilitate the survival of *Chlamydia* in the cell, yet only a few Incs have been characterized to date. The IncA protein of *C. trachomatis* is the best studied: it is capable of oligomerization (Delevoye et al., 2004) and is involved in the fusion of inclusions present in the same cell (Suchland et al., 2000). Delevoye et al. (2008) showed that IncA interacts with eukaryotic proteins called SNAREs (soluble N-ethylmaleimide-sensitive factor attachment protein receptors) that catalyze the reaction of membrane fusion during intracellular vesicular transport. IncG interacts with the host cell protein 14-3-3β, a component of signaling pathways (Scidmore and Hackstadt, 2001), and was shown to co-localize with Rab family proteins, specifically Rab11 (Rzomp et al., 2003), whereas IncD (from *C. trachomatis*) was found to associate with CERT (Ceramide Transport) proteins (Derré et al., 2011; Agaisse and Derré, 2014). Mital et al. (2010) showed that different Src kinases are recruited to the chlamydial inclusion and co-localized with several chlamydial inclusion membrane proteins. One function of Incs may be to interact with host cell components. However, many Incs are small proteins with limited cytoplasmically-exposed domains. Thus, we hypothesize that an alternative function of some of these Incs may be to facilitate interactions between other Incs. The resulting multimolecular complexes of Incs and host cell proteins may have a critical role in various processes that favor chlamydial growth.

In this study, we characterized interactions between different putative Inc proteins of *C. trachomatis* using a bacterial two-hybrid method (BACTH, bacterial adenylate cyclase-based two-hybrid) based on the reconstitution of a cyclic AMP (cAMP) signaling cascade in *Escherichia coli* (Karimova et al., 1998). In the BACTH system, two proteins of interest are fused to two complementary fragments from the catalytic domain of the adenylate cyclase of *Bordetella pertussis*, T25 and T18, and co-expressed in an *E. coli* Δ*cya* strain (i.e., lacking endogenous adenylate cyclase). When physically separated, the T25 and T18 fragments are inactive. Interaction between two hybrid proteins results in functional complementation between the T25 and T18 fragments, which restores the synthesis of cAMP and triggers the expression of catabolite genes (e.g., lactose operon or maltose regulon). As the interaction events can be spatially separated from the transcriptional readout, the BACTH system is particularly suitable to analyze *in vivo* interactions between intrinsic membrane proteins as exemplified in many studies (Karimova et al., 2005; Battesti and Bouveret, 2012; Ouellette et al., 2014a,b).

Here, we first verified that the BACTH system was appropriate to study the interaction properties of Inc proteins using the IncA protein as a model. We then analyzed a subset of Inc proteins and identified novel homo- and heterotypic interactions between these components. Our results suggest that the various Inc proteins assemble in the inclusion membrane to form supramolecular complexes.

## Materials and methods

### Strains and medium

The *E. coli* strain XL1-Blue (Stratagene, Santa Clara, CA) was used for all routine cloning experiments. Bacteria were routinely grown at 30°C in LB broth supplemented with appropriate antibiotics (ampicillin at 100 μg/mL, spectinomycin or kanamycin at 50 μg/mL). BACTH analyses were performed with *E. coli* Δ*cya* strain DHT1 (Dautin et al., 2000) (Table S1).

### Plasmid constructions

Standard protocols for molecular cloning, PCR (oligonucleotides are listed in Table S3), DNA analysis, and transformation were used (Sambrook and Russell, 2001). We used the Gateway^®^ technology of Invitrogen (Life Technologies, France) to clone the genes of interest into the BACTH-Gateway destination vectors, pST25-DEST and pUT18C-DEST (Ouellette et al., 2014a).

A collection of 280 *Chlamydia trachomatis* (serovar D/UW-3/CX) genes cloned into the Invitrogen pDONR221 vector was obtained from the Pathogen Functional Genomic Resource Center (PFGRC). This library contains 48 of the 59 putative *inc* genes predicted by Dehoux et al. (2011). Other putative *inc* genes not present within this PFGRC library were PCR amplified from genomic DNA of *C. trachomatis* L2 using appropriate primers encoding attB sites (Table S4). BP recombination reactions were performed to clone the attB-flanked PCR products into the pDONR™221 plasmid, following the manufacturer's guidelines. Briefly, 150 ng of the pDONR™221 plasmid was mixed with 150 ng of purified PCR products into 8 μL of TE buffer, and 2 μL of the BP Clonase™ II enzyme was added. The BP reaction was incubated for 2 h at room temperature. Proteinase K (1 μL—2 μg) was added to terminate the recombination reaction, and after 15 min of incubation at 37°C, half of the recombination reaction was used to transform 50 μL of *E. coli* XL1 competent cells. The transformants were selected on LB plates supplemented with 0.4% glucose and 50 μg/mL of kanamycin. The resulting plasmids (Table S2), encoding the gene of interest flanked by attL recombination sites, were verified by restriction analysis and sequencing.

The *inc* genes (flanked by attL recombination sites) were then transferred in a second step into the destination vectors, pST25-DEST, pUT18C-DEST, or pUT18C-f1-DEST, by the LR reaction, which was performed following the manufacturer's guidelines. The selection of recombinant bacteria was made on LB plates supplemented with 0.4% glucose and the appropriate antibiotic (spectinomycin or ampicillin). After sequencing, this recombinant vector was used for the BACTH complementation assays.

### BACTH complementation assays

For the BACTH test, the plasmids expressing the T25 and T18 fusions were transformed in DHT1 chemically competent cells (Sambrook and Russell, 2001), then washed with M63 and plated onto M63 minimum medium (containing 0.2% maltose, X-Gal (0.04 mg/mL), 0.5 mM IPTG, 0.04% casamino acids, 50 μg/ml spectinomycin and 100 μg/ml ampicillin), and incubated at 30°C for 3–4 days.

To measure the β-galactosidase activity of DHT1 bacteria expressing the fusion proteins, 8 clones were randomly selected from each series of transformants and resuspended in 300 μL of M63 medium containing maltose (0.2%), IPTG (0.5 mM), casamino acids (0.04%), ampicillin, and spectinomycin. These cultures were grown in 2.2 ml deep well 96-well plates overnight at 30°C. The next day, the cultures were diluted by adding 700 μL of M63 medium in each well. Then 200 μL were used to measure the optical density at 600 nm (OD_600nm_) using a TECAN spectrophotometer plate reader. 200 μL were transferred to a 1.2 ml polypropylene 96-well plate for measuring β-galactosidase activity. For this, the bacterial cells were permeabilized by adding 7 μL of 0.05% SDS and 10 μL of chloroform and mixing vigorously. Then, the plate was incubated for 1 h. For the enzymatic reaction, 20 μL of the permeabilized cells were added to 105 μL of PM2 buffer (PM2 medium supplemented with 0.125% ONPG and 58 μL of β-mercaptoethanol) in a microtiter plate. After 20 min, the enzymatic reaction was stopped by the addition of 50 μL of 1 M sodium carbonate Na_2_CO_3_to the mixture, and the optical density at 420 nm (OD_420nm_) was measured with the same apparatus as above. The enzymatic activity, A (in relative units), was calculated according to the following equation: A = 1000 × (OD_420_–OD_420_ in control well)/(OD_600_–OD_600_ in control well)/t (min) of incubation.

### Cell fractionation and detection of T18-IncA

The DHT1 containing the pUT18C-IncA and pST25-IncA recombinant plasmids were inoculated (1:100 dilution from an overnight culture) in 100 ml of LB medium containing glucose (0.4%) and ampicillin (100 mg/l). After 3 h incubation at 30°C, 50 μM of IPTG was added to induce expression of protein fusions. After 3 h of incubation at 30°C, the bacteria were harvested by centrifugation at 3000 g for 15 min at 4°C. Cell pellets were then resuspended in 2.5 mL of buffer I (10 mM Tris-HCl, 150 mM NaCl pH 8 supplemented with protease inhibitor cocktail from Roche Pharmaceuticals). The bacteria were disrupted by sonication and 500 μl (corresponding to the total lysate) was stored at −20°C. The remaining 2 mL were centrifuged 15 min at 3000 g to eliminate the debris and non-lysed cells. The supernatant was then centrifuged at 100,000 g for 1 h at 4°C. The supernatant, representing the soluble fraction of the cell (cytoplasm), was saved (at −20°C) while the pellet was resuspended in 500 μl of buffer I to which 50 μl of n-dodecyl-β-D-maltopyranoside (10% DDM) were added. After 1 h of incubation at 4°C, the membrane proteins solubilized in DDM were separated from the insoluble material by ultracentrifugation at 100,000 g for 30 min at 4°C. The insoluble fraction was resuspended in buffer I (500 μl). The three fractions were loaded on an SDS-PAGE (polyacrylamide gel electrophoresis) gel and transferred to a PVDF membrane. For this 30 μl of each fraction were mixed with 10 μl of 4 × NuPAGE^®^ LDS sample buffer (Invitrogen), heated 15 min at 95°C and then loaded on a SDS NuPAGE^®^ Novex^®^ 4-12% Bis-Tris gel (Invitrogen). After electrophoresis, the proteins were transferred onto a PVDF membrane using the iBlotTM Dry Blotting System (Invitrogen). Then, the membrane was blocked with a solution of milk powder (5%) in TTBS buffer (50 mM Tris-HCl pH 8.0, 0.15 M NaCl and 0.1% Tween20) for 1 h, rinsed twice with TTBS buffer, and finally incubated with a mouse monoclonal antibody 3D1 (Santa Cruz Biotechnology; sc-13582; it specifically recognizes the T18 fragment), diluted 1:1000 in TTBS buffer containing 1% milk powder and 10% glycerol. After 1 h of incubation, the membrane was again rinsed two times in TTBS buffer and then incubated with secondary antibody anti-mouse conjugated to peroxidase HRP (horseradish peroxidase) diluted 1:5000. After 1 h of incubation the membrane was again rinsed 5 times in TTBS buffer and the peroxidase activity was revealed by chemiluminescence using the ECL-plus kit (Amersham Biosciences) and exposed to film.

### Chlamydial organisms and cell culture

*Chlamydia trachomatis* serovar L2 Ebs were harvested from infected HeLa cell cultures at 37°C with 5% CO_2_, and purified by discontinuous density gradient centrifugation in Renografin (Bracco Diagnostics, Princeton, NJ). They were titrated for infectivity by determining inclusion forming units (IFU). HeLa cells were routinely cultivated at 37°C with 5% CO_2_ in IMDM (with glutamax) supplemented with 10% FBS (all from Invitrogen, Carlsbad, CA).

HeLa cells were plated in 6-well culture plates at a density of 1 × 10^6^ cells per well. In a subset of wells, cells were plated onto glass coverslips for immunofluorescence microscopy to monitor infection. Approximately 18 h later, confluent cell monolayers were rinsed with Hank's balanced salt solution (HBSS; Invitrogen), and, fresh medium containing 1 μg/mL cycloheximide was added to each well. *C. trachomatis* L2 was added to each well at a multiplicity of infection of 1.

### Quantification of transcripts by RT-qPCR

Quantitative transcriptional assays for the indicated *inc* genes were performed as described previously (Ouellette et al., 2014b). Briefly, total RNA was collected from *C. trachomatis* L2 infected HeLa cells at the indicated times using Trizol (Invitrogen) and treated with Turbo DNAfree (Ambion, Life Technologies) to remove contaminating DNA, according to the manufacturer's guidelines. One μg DNA-free RNA was reverse-transcribed with random nonamers (New England Biolabs, Ipswich, MA) using SuperScript III RT (Invitrogen) according to the manufacturer's instructions. Equal volumes of cDNA were used in qPCR reactions with SYBR Green (Quanta Biosciences, Gaithersburg, MD) and measured on an ABI 7300 system (Applied Biosystems, Life Technologies). Duplicate DNA samples were collected from the same experiment using Dneasy Tissue kit (Qiagen). Chlamydial genomes were quantified from equal amounts of total DNA by qPCR as above and used to normalize transcript data as described (Ouellette et al., 2005, 2006).

## Results

### Detection of IncA oligomerization by the bacterial two-hybrid system

**Figure 1:**
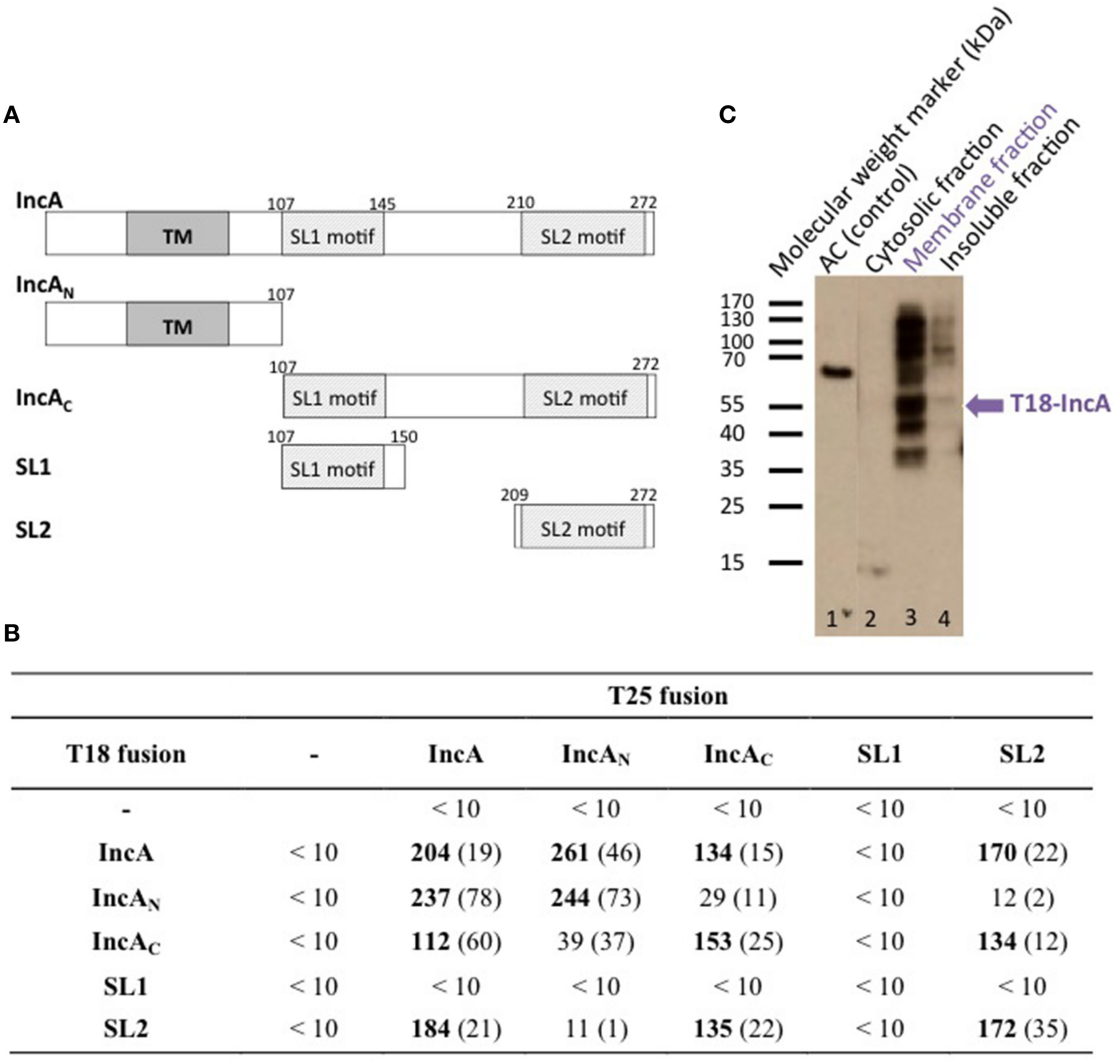
**BACTH analysis of *C. trachomatis* IncA and its sub-domains interactions. (A)** Schematic representation of the different domains of IncA (Ct119), with numbers indicating the amino acid residues. TM designates the transmembrane domain and SL1 and SL2 the two SNARE-like motifs. **(B)** The β-galactosidase activity of DHT1 co-expressing the indicated fusion proteins was measured in liquid cultures as described in “Materials and Methods.” The reported values, expressed in relative units (RU), correspond to the average obtained from eight clones tested for each interaction with the standard deviation given in parentheses. The “-” corresponds to the empty vectors. **(C)** Western blot analysis of the subcellular localization of the T18-IncA protein in *E. coli* DHT1. Exponentially growing DHT1 cells, co-expressing the T25-IncA and T18-IncA fusion proteins, were collected, lysed by sonication, and fractionated by ultracentrifugation (see Material and Methods). Proteins from the soluble (2), membrane (3) or insoluble (4) fractions were separated by gel electrophoresis on a 12% SDS-gel, transferred onto a PVDF membrane, and revealed with an anti-T18 monoclonal antibody (3D1). Positions of molecular weight markers (in kDa) are indicated on the left of the Figure while the expected position of T18-IncA is indicated by a blue arrow. In lane 1, a polypeptide corresponding to a 65 kDa fragment of CyaA adenylate cyclase (AC65) was run as a positive control for the anti-T18 antibody detection.

To determine whether the bacterial two-hybrid (BACTH) system (Karimova et al., 1998) could be used to characterize the interactions between Inc proteins, we first examined the oligomerization properties of the IncA protein. For this, the *incA* coding region was amplified by PCR from the chromosomal DNA of *C. trachomatis* serovar L2 and cloned into the BACTH vectors pST25 and pUT18C (Figure 1A; Table 1) to yield plasmids, pST25-*incA* and pUT18C-*incA*, that express the fusion proteins T25-IncA and T18-IncA, respectively. All Inc proteins and protein domains were fused to the C-terminus of T25 or T18. These plasmids were then co-transformed into the *E. coli* DHT1 Δ*cya* strain, together with various plasmids encoding fusions with unrelated chlamydial proteins or empty vectors, to serve as negative controls. The transformed bacteria were plated on selective medium, and β-galactosidase activity for each interaction was measured from eight randomly selected colonies. As shown in Figure 1B, DHT1 bacteria that co-expressed the T25 and T18 proteins fused to full-length IncA showed high levels of β-galactosidase activity (204 ± 19 relative units, RU), about 20 times higher than that of bacteria harboring control plasmids (<10 RU). This indicates that the two fusion proteins, T25-IncA and T18-IncA, efficiently associated in *E. coli*.

**Table 1:** **BACTH analysis of interactions between IncB, Ct101, Ct222, and Ct850**.

**T25 fusions**				
**T18 fusions**	**IncB**	**Ct101**	**Ct222**	**Ct850**
–	10 (5)	10 (5)	10 (5)	10 (5)
IncB	8 (4)	6 (1)	10 (2)	8 (1)
Ct101	7 (6)	8 (1)	14 (10)	8 (2)
Ct222	9 (1)	9 (1)	**317** (116)	**92** (18)
Ct850	5 (1)	9 (7)	**151** (88)	32 (17)

The β-galactosidase activity of the DHT1 bacteria, co-expressing the indicated hybrid proteins, was measured as described in “Materials and Methods.” The reported values, expressed in relative units (RU), correspond to the average obtained from eight clones tested for each interaction with standard deviation given in parentheses. positive interactions are in bold.

### Localization of IncA fusion protein in *E. coli*

The Inc proteins are bacterial membrane proteins that localize *in vivo* in the chlamydial inclusion membrane, which is derived from a eukaryotic membrane. To verify that the IncA hybrid proteins properly inserted into the *E. coli* membrane when tested in the BACTH assay, we used a cell fractionation method (Karimova et al., 2009). DHT1 cells co-expressing T25-IncA and T18-IncA, were grown in LB, lysed by sonication, and fractionated by differential centrifugation (see Materials and Methods). The proteins contained in the different fractions (i.e., cytoplasmic, membrane, and insoluble fractions) were separated by polyacrylamide gel electrophoresis and transferred onto a PVDF membrane. The T18-IncA fusion protein was detected in the different fractions with a monoclonal antibody (3D1) directed against the T18 fragment. The T18-IncA fusion protein (molecular weight of ≈52kDa) was predominantly localized in the bacterial membrane fraction (Figure 1C). Several additional bands were detected on the blot, with the higher bands likely corresponding to oligomeric species not dissociated by SDS (as frequently seen with oligomeric membrane proteins), while the lower ones may correspond to degraded forms of T18-IncA. These results thus confirmed that the T18-IncA fusion was indeed associated with the *E. coli* membrane when expressed in DHT1.

### Characterization of the oligomerization properties of IncA sub domains

IncA contains two distinct regions, an N-terminal region, IncA_N_, that harbors two transmembrane helices TM predicted to insert into the membrane of the inclusion, and a C-terminal region localized in the cytoplasm, IncA_C_, previously shown by Delevoye et al. (2008) to contribute to the oligomerization of IncA. We constructed BACTH plasmids encoding these two distinct functional sub-domains of IncA fused to the T25 or T18 fragments (Figure 1A) to examine their interaction properties. As shown in Figure 1B, BACTH assays indicate that each of the functional domains independently oligomerized and also interacted with the full-length IncA protein, while IncA_N_ and IncA_C_ did not interact. Hence, the BACTH system confirmed the report of Delevoye et al. (2008) and further revealed that the IncA transmembrane domain, in addition to IncA_C_, also exhibits an intrinsic capacity to oligomerize.

Delevoye et al. (2008) further identified in IncA_C_ two segments (SL1 and SL2) exhibiting some similarity to eukaryotic SNARE (Soluble N-ethylmaleimide-sensitive-factor Attachment protein Receptor) motifs. SNAREs play an important role in the fusion of eukaryotic intracellular vesicles by assembling stable four-helix bundles to allow fusion of two distinct compartments (Jahn and Scheller, 2006). Delevoye et al. (2008) have shown that the SNARE motifs of IncA are important for interactions with eukaryotic SNAREs and with IncA itself, possibly facilitating the fusion of inclusions. To determine which of the two “SNARE-like” motifs, SL1 or SL2, may be involved in the homotypic interaction of IncA, these individual motifs were fused to T25 or T18 and tested in BACTH assays (Figure 1). While no interaction was detected with SL1, we found that the SL2 motif interacted with itself, IncA_C_, and the full-length IncA, but not with IncA_N_, in agreement with our above results. These data are thus consistent with the involvement of SL2 in the homotypic interaction of IncA (Delevoye et al., 2008).

In sum, this BACTH study confirmed the interaction properties of IncA previously established by Delevoye et al. (2008) and further revealed that the transmembrane region contributes to the oligomerization of IncA, independently of the IncA cytoplasmic domain. Importantly, our results validate the BACTH system as being suitable for studying the interaction properties of the *C. trachomatis* inclusion membrane proteins.

### BACTH analysis of interactions between IncB, Ct101, Ct222, and Ct850

**Figure 2:**
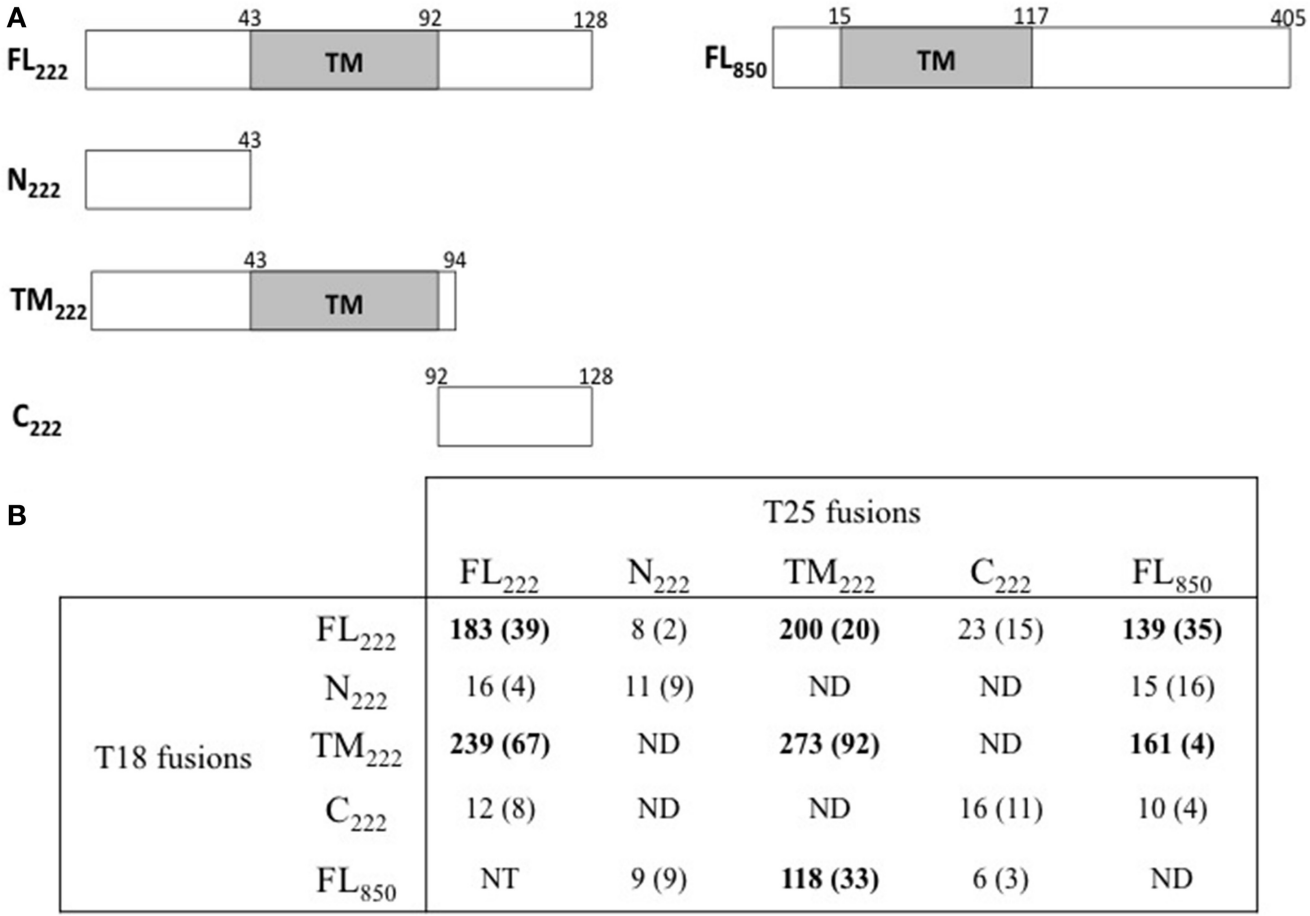
**BACTH analysis of interactions between Ct222 sub-domains. (A)** Schematic representation of the different domains of Ct222 with numbers indicating the amino acid residues. FL means full-length proteins. **(B)** BACTH interaction assays between the different domains of Ct222 and Ct850 are listed in this table and expressed in relative units (RU). All Inc proteins were fused to the C-terminus of T25 or T18. NT: not tested. ND: not detected as the corresponding transformants formed only white colonies on indicator plates and therefore should display only background levels of β-galactosidase activity (<10 RU).

Mital et al. (2010) have previously shown by immunofluorescence that IncB co-localized at the inclusion membrane with three putative Incs, Ct101, Ct222, and Ct850. They further showed that only Ct222 and Ct850 co-immunoprecipitated while IncB and Ct101 did not. To verify these interactions, we constructed BACTH vectors encoding each of these four genes using a Gateway^®^-compatible system, BACTH_GW_ (Ouellette et al., 2014a), and the resulting recombinant plasmids were then tested in BACTH assays. As shown in Table 1, our results demonstrated a strong interaction between Ct222 and Ct850 but not with IncB and Ct101, in agreement with co-IP studies of Mital et al. (2010). In addition, we also found that Ct222 could efficiently oligomerize. To further delineate the polypeptide regions involved in the interaction of Ct222 with itself and Ct850, the different regions corresponding to the N-terminal (N_222_), the transmembrane hairpin domain (TM_222_), or C-terminal region (C_222_) of Ct222 were amplified and cloned into BACTH vectors that were then tested in BACTH assays. We found that the TM domain of Ct222 was sufficient to mediate its homo-oligomerization as well as its interaction with Ct850 whereas no interactions were detected with the cytosolic domains, N_222_ or C_222_ (Figure 2). Our data thus indicate that the association of Ct222 with itself and Ct850 is mediated by its TM domain while its cytosolic domains may remain available for interacting with putative additional partners from the bacterium or the eukaryotic host.

### Homo-oligomerization of a subset of Inc proteins

**Figure 3:**
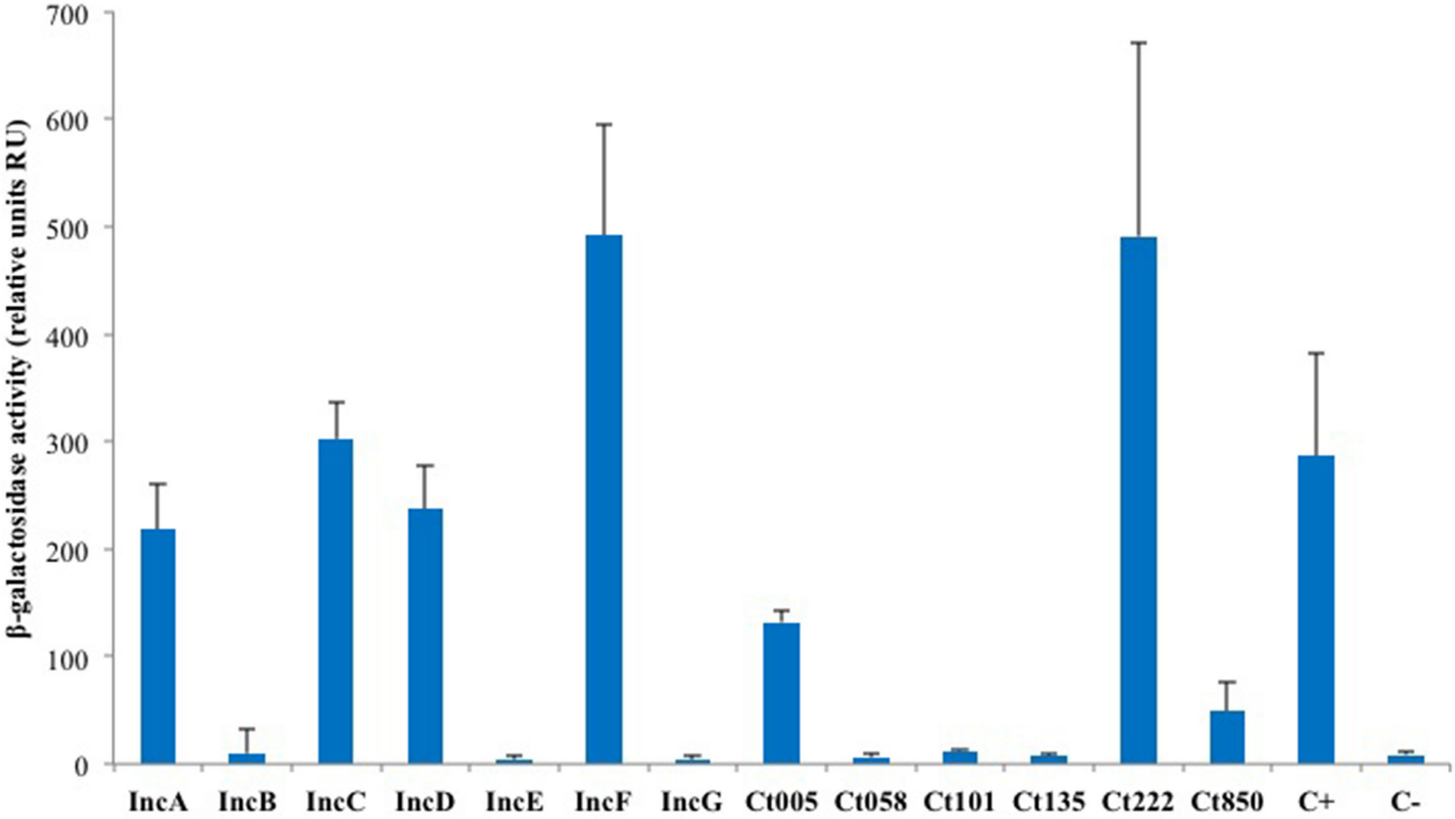
**BACTH analysis of the homotypic interactions of *C. trachomatis* Incs**. DHT1 bacteria were co-transformed with plasmids expressing the T25 and T18 fusions to the indicated *inc* proteins. The β-galactosidase activity values correspond to the average obtained from eight independent colonies. The positive control C+ corresponds to the interaction between the T25-FtsW and T18-FtsI proteins of *E. coli* (Karimova et al., 1998), while the negative control C- to cells harboring the empty vectors pST25 and pUT18C.

As IncA and Ct222 were found to homo-oligomerize, we explored more systematically the ability of a subset of Inc proteins to self-interact in BACTH assays. For this initial analysis, we selected the annotated Inc proteins (IncA to IncG), the four Incs studied by Mital et al. (2010) and a few others randomly chosen from the library we obtained from the Pathogen Functional Genomic Resource Center (containing *inc* genes from *Chlamydia trachomatis* serovar D). DHT1 cells were co-transformed with plasmids expressing the T25 and T18 fused to the same Inc proteins, and the β-galactosidase activities in liquid cultures of the transformed bacteria were measured to quantify the interaction. The results of these experiments, shown in Figure 3, revealed that IncC, IncD, IncF, and Ct005 also oligomerized as seen for IncA and Ct222. Thus, the ability to establish homotypic interactions appears to be common among Inc proteins.

### Interaction network among Inc proteins

To analyze more broadly the interactions between the Inc proteins, we carried out a systematic BACTH screen of interactions among a subset of 20 known or predicted Inc proteins (Li et al., 2008; Dehoux et al., 2011). For this, two complementary approaches were used. In a first approach, the DHT1 cells were co-transformed with a given pST25-*inc* plasmid (“bait” proteins in Table 2) and an equimolar mixture (“pool”) of each pUT18C-*inc* plasmids, and the transformed bacteria were plated onto selective medium for 4–6 days of incubation. Blue colonies, corresponding to Cya^+^ bacteria that expressed interacting hybrid proteins, were randomly picked and their pUT18C-*inc* plasmids were identified by sequencing. In a second approach, the DHT1 cells were co-transformed with pairwise combinations of pST25-*inc* and pUT18C-*inc* plasmids, plated on selective medium and, after 3–4 days of incubation, eight colonies from each combination were grown in liquid culture to measure the β-galactosidase activity as above. As shown in Table 2, this screen revealed numerous novel interactions between the subset of Inc proteins tested. Remarkably, several Inc proteins, like IncF, IncD, and Ct222, appeared to interact with many other Incs and may thus represent primary proteins that form interaction nodes within the inclusion membrane. In contrast, we could not detect any interaction for IncB, IncE, Ct101, Ct134, Ct135, and Ct227. This could be due to the fact that the BACTH system might not be appropriate for these particular proteins or alternatively, that these Incs had no specific partner in the subset of Inc proteins tested. Altogether, our study with the BACTH system revealed numerous novel interactions between the Inc proteins of *Chlamydia*. These results suggest that these proteins assemble into specific multimolecular complexes within the inclusion membrane.

**Table 2:** **BACTH analysis of hetero-oligomerization of the Inc proteins**.

**Bait protein T25 fusion**	**MW^a^ (Da)**	**Developmental expression^b^**	**Prey protein T18 fusion**
Ct005	39537	early	**Ct005**, IncA, IncF
Ct058	40072	Mid	IncD
Ct101	17645	Mid	NO
Ct115 incD	14912	early	**IncD**, Ct058, Ct222
Ct116 incE	13539	early	NO
Ct117 incF	10420	early	**IncF**, IncA, IncC, IncD, IncG, Ct005, Ct058, Ct249, Ct850
Ct118 incG	17389	early	IncD
Ct119 incA	30313	Mid	**IncA**, IncC, IncD, IncF, Ct005
Ct134	15104	NT	NO
Ct135	38477	early	NO
Ct222	13915	Mid	**Ct222**, IncD, Ct223, Ct224, Ct850
Ct223	29475	Mid	**Ct223**
Ct225	13264	Mid	**Ct225**
Ct227	14155	Mid	NO
Ct229	23422	early	IncD, Ct222, Ct223
Ct232 incB	12237	early	NO
Ct233 incC	18418	early	**IncC**, IncA
Ct249	12210	early	**Ct249**, IncF
Ct813	29570	Mid	**Ct813**
Ct850	45822	Mid	IncF, Ct222

The interactions identified with the BACTH system are listed in this table. All Inc proteins were fused to the C-terminus of T25 or T18. An interaction corresponds at a β-galactosidase activity of the DHT1 bacteria at least 5 times superior to the negative control (see Table S5). NO means that we did not find any blue clones (no interaction detected) on the plate. The homotypic interactions are in bold.

a Deduced from protein sequence.

b Developmental expression pattern as deduced from qPCR analysis.

### Transcriptional profiling of candidate Inc genes

**Figure 4:**
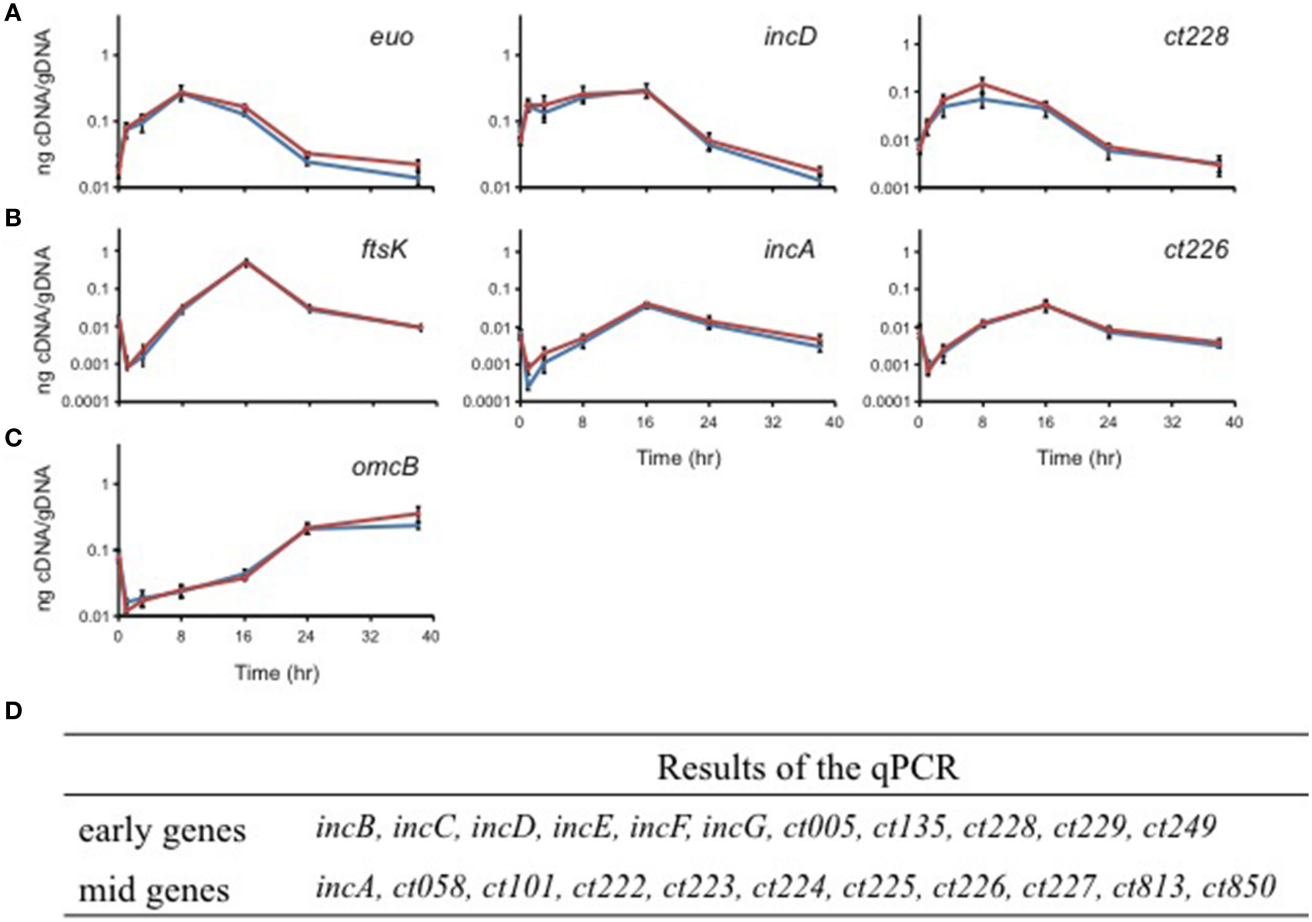
**Quantitative transcriptional analysis of *inc* genes**. The developmental transcription profile of various *inc* genes was assessed. Total RNA was isolated over a time course of infection with *C. trachomatis* L2, and transcription of individual *inc* genes was quantified by RT-qPCR normalized to genomic DNA (ng cDNA/DNA). **(A)** indicates early genes with *euo* as a marker. **(B)** indicates mid-cycle genes with *ftsK* as a marker. **(C)** indicates late genes with *omcB* as a marker. No *inc* genes were transcribed late. **(D)** Summary of the transcriptional profiles of *inc* genes (see also Figure 5).

*Chlamydia* is a developmentally regulated bacterium, and its transcriptional activity can be broadly divided into early, mid, and late stages corresponding to EB-to-RB differentiation (e.g., *euo*; Wichlan and Hatch, 1993), RB growth (e.g., *ftsK*; Ouellette et al., 2012), and RB-to-EB transition (e.g., *omcB*; Fahr et al., 1995), respectively. As a means of understanding the significance of interactions between Incs in the context of infection, we quantitatively measured the transcription of the indicated *inc* genes to accurately determine their developmental expression pattern. In agreement with prior publications (Scidmore-Carlson et al., 1999; Shaw et al., 2000; Belland et al., 2003), *incB-G* are all early stage genes with a pattern of expression similar to *euo*. To this list, we add *ct005, ct135, ct228, ct229, ct288, ct249*, and *ct813*. The expression pattern of *incA* is mid-cycle (Hackstadt et al., 1999), similar to *ftsK*, and, to this list, we add *ct058, ct101, ct222-227*, and *ct850* (Figure 4 and Table 2).

## Discussion

The inclusion membrane proteins of *Chlamydia trachomatis* likely play a key role in the molecular and cellular interactions between the pathogen and the host (Betts et al., 2009). However, the majority of these proteins remain uncharacterized for two main reasons. Firstly, as intrinsic membrane proteins, Incs are difficult to characterize by biochemical analysis. Secondly, *Chlamydia* is difficult to manipulate genetically in spite of recent successes in transforming the bacterium (Wang et al., 2011). Thus, alternative methods are needed for studying these important proteins.

We hypothesized that Inc proteins assemble into the inclusion membrane to form different multi-protein complexes that may then interact with host proteins to modify the host cell physiology. Thus, to explore the associations between the Inc proteins, we employed a bacterial two-hybrid (BACTH) system, based on the reconstitution of a cAMP signaling cascade in an *E. coli* Δ*cya* strain, that has been widely used to study interactions between intrinsic membrane proteins (Karimova et al., 2005; Battesti and Bouveret, 2012; Ouellette et al., 2014a,b). We first verified that the BACTH system was able to detect oligomerization of IncA, the best characterized Inc protein (Delevoye et al., 2004, 2008). BACTH analysis revealed that IncA oligomerized efficiently in *E. coli* and confirmed that the cytoplasmic region, that contains SNARE-like motifs, could independently oligomerize, in good agreement with the previous study of Delevoye et al. (2008). More importantly, we showed for the first time that the N-terminal region that encompasses the transmembrane segments of IncA was also able to oligomerize. This finding suggests a model for IncA assembly that has implications for its function. In *C. trachomatis*, IncA is known to promote the fusion of multiple inclusions that result from multiple EBs infecting a same cell, each giving rise to an distinct/individual inclusion (Hackstadt et al., 1999; Suchland et al., 2000). Our data suggest that IncA may oligomerize in the membrane of a first inclusion via its transmembrane region while its cytoplasmic, SNARE-like (SL) motifs could interact with cognate domains from similar IncA oligomers inserted within a separate, distinct inclusion. This oligomerization of IncA through its transmembrane region might facilitate the formation of putative parallel four helix bundles by the SNARE-like structures. Multiple interactions between clustered SL motifs may also synergize to favor close apposition of the two inclusion membranes as a first step toward their fusion. The clustering of SNARE-like (SL) motifs within IncA oligomers may also contribute to the specific recruitment of host SNAREs such as Vamp3, Vamp7, or Vamp8 to the inclusion membrane as shown by Delevoye et al. (2008).

The BACTH system was then used to analyze the association of four Inc proteins, IncB, Ct101, Ct222, and Ct850, that were previously shown by Mital et al. (2010) by immunofluorescence techniques to co-localize at the inclusion membrane. Among these four Incs, only Ct222 and Ct850 were suggested to directly interact as indicated by co-immunoprecipitation (Mital et al., 2010). Importantly, we also detected a strong interaction between Ct222 and Ct850 in the BACTH assay thus confirming that they are able to physically associate independently of any other chlamydial or eukaryotic component. We further demonstrated that the transmembrane domain of Ct222 was directly involved in this association suggesting, again, that interactions within the inclusion membrane may be critical for the assembly of multi-Inc complexes. In contrast, we did not detect any interactions with IncB or Ct101, in accordance with Mital et al. (2010) results. In fact, no interacting partners were found for IncB and Ct101, nor for other Inc proteins like Ct134, Ct135, and Ct227 among a subset of 20 Inc proteins tested. Obviously, we cannot rule out the possibility that these Inc proteins were not properly folded in the inner membrane of *E. coli*, and therefore nonfunctional for interacting in bacterial two-hybrid assays. Alternatively, their association into a multi-molecular complex may require a specific lipid composition found only in the inclusion membrane or may require additional chlamydial or eukaryotic components not present in our bacterial assays. This latter possibility is the most probable given the lack of co-immunoprecipitation of IncB and Ct101 with Ct222 and Ct850 reported by Mital et al. (2010). A more exhaustive BACTH screen may help to identify these putative additional chlamydial components required for their assembly in multi-protein complexes. It may also be worthwhile to construct eukaryotic BACTH libraries to extend this screen to host cell components. Mital et al. (2010) reported that two members of the Src kinase family were colocalized in their active form with these four Inc proteins, although they did not further examine whether these kinases might be involved in the clustering of these Incs within the inclusion membrane.

**Figure 5:**
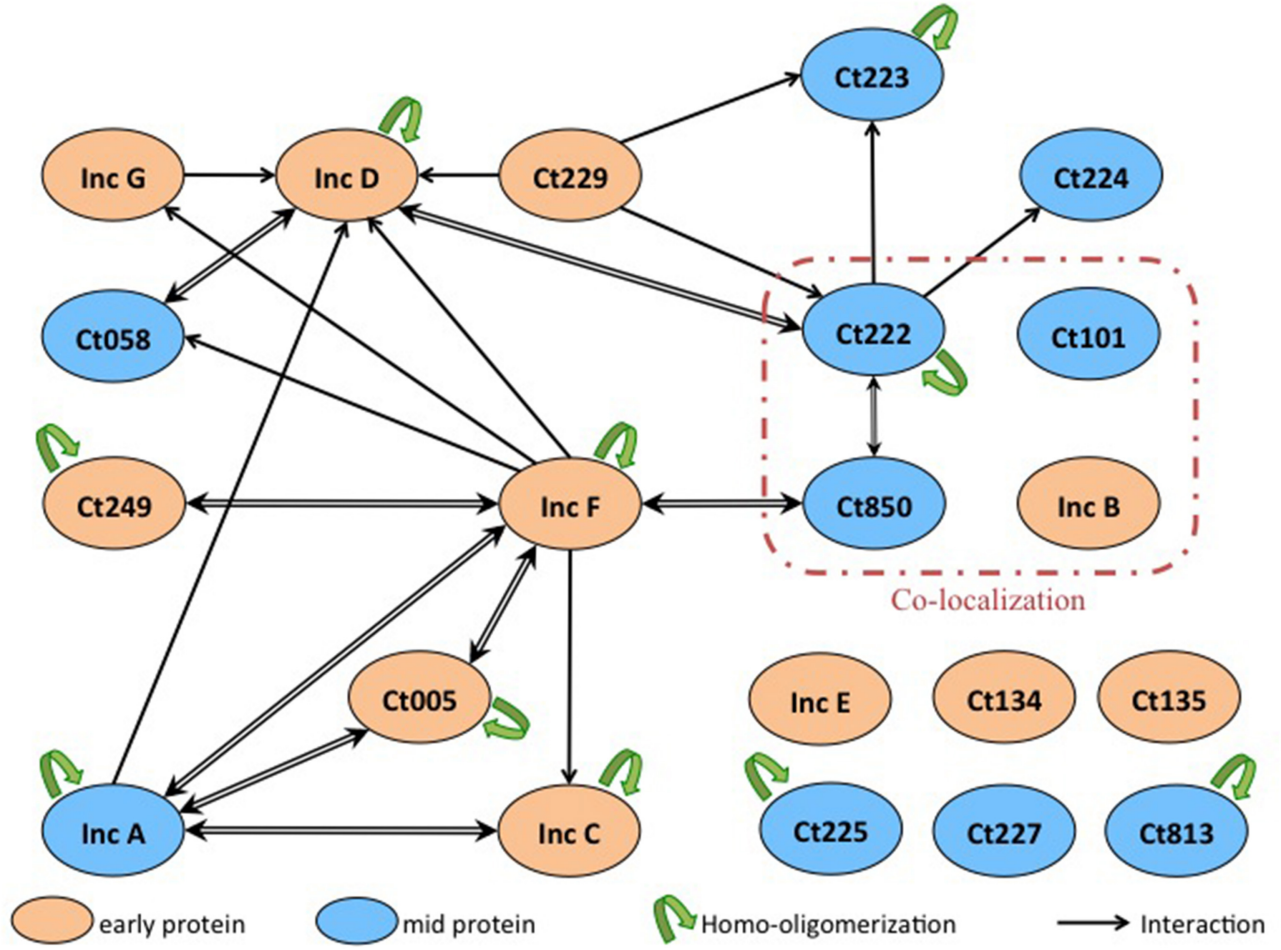
**Interaction network among Inc proteins**. Schematic overview of the interactions between the Inc proteins that are colored according to their developmental expression pattern: orange for early genes and blue for mid genes. The interactions identified in this work are indicated with black arrows (the arrow pointing toward the T18-fusion indicates an interaction with a corresponding T25-fusion), and the homo-oligomerizations are shown by green arrows. Proteins reported to co-localize in Mital et al.'s experiment are surrounded with a red line.

Finally, we identified numerous novel homo- and heterotypic interactions between the subset of Inc proteins that we tested. Two notable results were obtained. Firstly, we identified several Incs in addition to IncA capable of homo-oligomerization as IncC, IncD, IncF, Ct005, and Ct222 also displayed self-interacting properties. Secondly, our studies of the heterotypic interactions revealed that several small Incs, with limited predicted cytosolically-exposed domains, notably IncF and Ct222, were able to interact with multiple partners. These proteins may thus serve as critical interaction nodes to organize a dense network of Incs within the inclusion membrane, as depicted in Figure 5. The oligomerization of Inc proteins reinforces the hypothesis that Incs may function as multi-protein complexes. The present data have been obtained with the Inc proteins from *C. trachomatis* serovar D or L2 but our results may likely be similarly pertinent for other *Chlamydia* serovars or species.

Incs are commonly thought to interact with host cell components to facilitate the organism's subversion of the host cell. However, approximately one quarter of all putative Incs encode a short cytoplasmic domain of roughly 20 amino acids with no structural domains thus how such small proteins could interact with host cell components is unclear. A more likely scenario is that these small Incs serve an alternate function to create a scaffold within the inclusion membrane to facilitate assembly of specific multi-protein complexes that in turn interact with host cell component(s). For example, IncF consists of 104 amino acids of which 38 N-terminal amino acids encoding the signal sequence for the type III system and 12 C-terminal amino acids may be localized in the host cell cytoplasm. This suggests that IncF or other small Incs interact with other Inc proteins by their transmembrane domain. Remarkably, IncF seems to be at the center of many interactions. We hypothesize that the small Inc proteins, like IncF, oligomerize at the inclusion membrane and recruit other Incs involved in interactions with host cell components, such as IncG that interacts with 14-3-3β or Rab11 (Scidmore and Hackstadt, 2001; Rzomp et al., 2003). Thus, IncF could act as an interaction node for Inc proteins.

Typically referred to as a parasitophorous vacuole residing in the exocytic pathway, the inclusion may be more accurately envisioned as a pathogen-specified parasitic organelle that dynamically interacts with various host cell compartments (Moore and Ouellette, 2014). In this context, the Inc proteins serve both as markers of the organelle and key constituents of the inclusion membrane. Given that individual Inc proteins are neither expressed at the same level nor at the same time during the developmental cycle of *Chlamydia* (see Figures 4, 5), we can infer that different Incs may serve different purposes at different times. For example, IncD, IncF, and IncG are expressed early in the developmental cycle and they interact with many other Inc proteins, like Ct058 or Ct850, which are expressed later during the cycle. In support of this, IncG can be detected at later times during infection and appears stable since it remains associated with the inclusion even after 24 h of treatment with chloramphenicol (Moore et al., 2011). Likewise, IncB is expressed early and co-localizes with Ct101, Ct222, and Ct850, each of which is expressed later in the developmental cycle, in the study by Mital et al. (2010). Indeed, chlamydial proteins may be generally stable (Ouellette et al., 2006) thus it is probable that Incs expressed early are present later. However, it remains possible that the interaction between an early and a late protein never occurs in the developmental cycle of *Chlamydia* because the proteins never encounter each other, in which case the interactions detected by BACTH assay would not be relevant.

In conclusion, our data support a model wherein different complexes can form throughout the *Chlamydia* developmental cycle to facilitate interactions with the host cell that promote the growth and development of the bacteria while having a minimal impact on the host cell. We hypothesize that these assemblies play an important role in the physiology of the bacterium and in its interaction with the host cell. The characterization of the interaction network between the Inc proteins and also with its eukaryotic partners will allow a better understanding of their function in the developmental cycle of *Chlamydia*.

## Author contributions

Emilie Gauliard, Scot P. Ouellette and Daniel Ladant designed experiments, analyzed data, and wrote the manuscript. Emilie Gauliard, Scot P. Ouellette, and Kelsey J. Rueden performed experiments.

### Conflict of interest statement

The authors declare that the research was conducted in the absence of any commercial or financial relationships that could be construed as a potential conflict of interest.
