# Supplementary material for: Characterization of interactions between inclusion membrane proteins from Chlamydia trachomatis
Source: Front Cell Infect Microbiol. 2015 Feb 11;5:13. doi: 10.3389/fcimb.2015.00013 (PMC4324299; doi:10.3389/fcimb.2015.00013)
Supplement: Supplementary file 1 [file Table1.DOCX]

**Table S1. *E. coli* strains and plasmids for cloning**

| Strain/plasmid | Relevant genotype | Source or reference |
| --- | --- | --- |
| XL1 | *F’::Tn10 proA+B+ lacI^q^ Δ(lacZ)M15, glnV44(AS), endA1, gyrA96, recA1, thi-1, hsdR17 lac* | Stratagene, Santa Clara, CA |
| DHT1 | F^-^ *glnV44* (AS) *recA1 endA1 gyrA96* (Nal^R^) *thi-1 hsdR17 spoT1 rfbD1 cya-854 ilv-691* ::Tn*10* (Tet^R^) | In the lab (Dautin *et al*., 2000) |
| pST25 | *aadA* P_lac_::*t25* | Ouellette *et al.,* 2014a |
| pUT18C | *bla* P_lac_::*t18* | Karimova *et al*., 2001 |
| pST25-DEST | *aadA* P_lac_::*t25-att*R1-[*cat ccdB*]-*att*R2 | Ouellette *et al*., 2014a |
| pUT18C-DEST | *bla* P_lac_::*t18-att*R1-[*cat ccdB*]-*att*R2 | Ouellette *et al.,* 2014a |
| pDONR221 | *aph* *att*P1-[*cat ccdB*]-*att*P2 | Invitrogen (Life Technologies) |
