# Supplementary material for: Characterization of interactions between inclusion membrane proteins from Chlamydia trachomatis
Source: Front Cell Infect Microbiol. 2015 Feb 11;5:13. doi: 10.3389/fcimb.2015.00013 (PMC4324299; doi:10.3389/fcimb.2015.00013)
Supplement: Supplementary file 2 [file Table2.DOCX]

**Table S2. Inc plasmids**

| gene | Entry plasmid | Source or reference | final plasmid | | |
| --- | --- | --- | --- | --- | --- |
|  |  |  | pST25 | pUT18C | |
| *ct005* | pENTR005 | This work | pST25-Ct005_GW_ | pUT18C-Ct005_GW_ | |
| *ct058* | pENTR058 | This work | pST25-Ct058_GW_ | pUT18C-Ct058_GW_ | |
| *ct101* | pENTR101 | PFGRC | pST25-Ct101_GW_ | pUT18C-Ct101_GW_ | |
| *ct115 incD* | pENTR115 | PFGRC | pST25-IncD_GW_ | pUT18C-IncD_GW_ | |
| *ct116 incE* | pENTR116 | PFGRC | pST25-IncE_GW_ | pUT18C-IncE_GW_ | |
| *ct117 incF* | pENTR117 | PFGRC | pST25-IncF_GW_ | pUT18C-IncF_GW_ | |
| *ct118 incG* | pENTR118 | This work | pST25-IncG_GW_ | pUT18C-IncG_GW_ | |
| *ct119 incA* | pENTR119 | PFGRC | pST25-IncA_GW_ | pUT18C-IncA_GW_ | |
| *ct134* | pENTR134 | PFGRC | pST25-Ct134_GW_ | - | |
| *ct135* | pENTR135 | This work | pST25-Ct135_GW_ | pUT18C-Ct135_GW_ | |
| *ct222* | pENTR222 | PFGRC | pST25-Ct222_GW_ | pUT18C-Ct222_GW_ | |
| *ct223* | pENTR223 | PFGRC | pST25-Ct223_GW_ | pUT18C-Ct223_GW_ | |
| *ct224* | pENTR224 | This work | - | pUT18C-Ct224_GW_ | |
| *ct225* | pENTR225 | PFGRC | pST25-Ct225_GW_ | pUT18C-Ct225_GW_ | |
| *ct227* | pENTR227 | PFGRC | pST25-Ct227_GW_ | pUT18C-Ct227_GW_ | |
| *ct229* | pENTR229 | PFGRC | pST25-Ct229_GW_ | - | |
| *ct232 incB* | pENTR232 | PFGRC | pST25-IncB_GW_ | pUT18C-IncB_GW_ | |
| *ct233 incC* | pENTR233 | This work | pST25-IncC_GW_ | pUT18C-IncC_GW_ | |
| *ct249* | pENTR249 | PFGRC | pST25-Ct249_GW_ | pUT18C-Ct249_GW_ | |
| *ct813* | pENTR813 | PFGRC | pST25-Ct813_GW_ | pUT18C-Ct813_GW_ | |
| *ct850* | pENTR850 | PFGRC | pST25-Ct850_GW_ | pUT18C-Ct850_GW_ |  |
