# Supplementary material for: Characterization of interactions between inclusion membrane proteins from Chlamydia trachomatis
Source: Front Cell Infect Microbiol. 2015 Feb 11;5:13. doi: 10.3389/fcimb.2015.00013 (PMC4324299; doi:10.3389/fcimb.2015.00013)
Supplement: Supplementary file 3 [file Table3.DOCX]

**Table S3. Oligonucleotides used in this study.**

The resulting PCR product was digested and inserted into the corresponding BamHI and KpnI sites of pST25 and pUT18C. The BamHI or KpnI site, for the forward or the reverse respectively, is underlined and the start or the end of the gene is bolded.

| Gene | Primer | Sequence (5’→3’) | Final plasmid |  |
| --- | --- | --- | --- | --- |
| *incA* Full Length | Forward  Reverse | ATTTTGGATCCT**ATG**ACAACGCCTACTCTA  ATATAGGTACCAA**GGA**GCTTTTTGTAGAGGG | pST25-IncA pUT18C-IncA | |
| *incA* N-terminal | Forward  Reverse | ATTTTGGATCCT**ATG**ACAACGCCTACTCTA  CCTCCGGTACCCG**ATT**AATTTCTTTTAGAGA | pST25-IncA_N_ pUT18C-IncA_N_ | |
| *incA* C-terminal | Forward  Reverse | CCGCCGGATCCT**TCT**CTAAAAGAAATTAAT  ATATAGGTACCAA**GGA**GCTTTTTGTAGAGGG | pST25-IncA_C_ pUT18C-IncA_C_ | |
| *incA* SL1 motif | Forward  Reverse | ATCTTGGATCCT**AAT**TTCATGCTGAGCGTT  ATCTCGGTACCAA**AAA**TCCTTGCAAACAAGA | pST25-IncA_SL1_  pUT18C-IncA_SL1_ | |
| *incA* SL2 motif | Forward  Reverse | ATCTTGGATCCT**AAT**TTCATGCTGAGCGTT  ATATAGGTACCAA**GGA**GCTTTTTGTAGAGGG | pST25-IncA_SL2_  pUT18C-IncA_SL2_ | |
| *ct222* FL_222_ | Forward  Reverse | AATATGGATCCT**CGT**TGCTGTTGTGTTCGTAC  ACATAGGTACCCC**TCA**GTGGAATACACTAATTG | pST25-Ct222_FL_  pUT18C- Ct222_FL_ | |
| *ct222* N_222_ | Forward  Reverse | AATATGGATCCT**CGT**TGCTGTTGTGTTCGTAC  CTAACGGTACCTC**TAC**AGACGCAATCTTTCTAC | pST25- Ct222_N_  pUT18C- Ct222_N_ | |
| *ct222* TM_222_ | Forward  Reverse | AATATGGATCCT**CGT**TGCTGTTGTGTTCGTAC  AGACAGGTACCGA**CAT**ACGCAAAAATAGTTTAT | pST25- Ct222_TM_  pUT18C-Ct222_TM_ | |
| *ct222* C_222_ | Forward  Reverse | TATATGGATCCA**TTG**CGTATGAAGCGAGTGTC  ACATAGGTACCCC**TCA**GTGGAATACACTAATTG | pST25- Ct222_C_  pUT18C- Ct222_C_ | |
| *ct850* FL_850_ | Forward  Reverse | TATATGGATCCT**GGA**TTCGGAACTGTGAGAGG  ATTAAGGTACCAC**TTA**CCGATTCTGGTTGTGAA | pST25- Ct850_FL_  pUT18C- Ct850_FL_ | |
