# Supplementary material for: Characterization of interactions between inclusion membrane proteins from Chlamydia trachomatis
Source: Front Cell Infect Microbiol. 2015 Feb 11;5:13. doi: 10.3389/fcimb.2015.00013 (PMC4324299; doi:10.3389/fcimb.2015.00013)
Supplement: Supplementary file 4 [file Table4.DOCX]

**Table S4. Oligonucleotides Gateway® used in this study.**

The different genes were amplified from *C. trachomatis* L2 genomic DNA with the primers mentioned above. The attB site is underlined and the start or the end of the gene is bolded. The resulting PCR product was transferred into the pDONR221™ by recombination (BP reaction).

| Gene | Primer | Sequence (5’→3’) | Final plasmid |
| --- | --- | --- | --- |
| *ct005* | Forward  Reverse | AATTAACAAGTTTGTACAAAAAAGCAGGCTTT**A**TGACTCCAGTAACACCAGTCCCTCCC CCGTACCACTTTGTACAAGAAAGCTGGGTT**T**TTACGAGAGGGTTTCTTCTTTTGAGA | pENTR005 |
| *ct058* | Forward  Reverse | TATGAACAAGTTTGTACAAAAAAGCAGGCTTT**A**TGTTTACGTCGCTGTCCGCAATACAG  CATATCCACTTTGTACAAGAAAGCTGGGTT**C**AAATTCACGGGTTGAGGGATGTTCGT | pENTR058 |
| *incG* | Forward  Reverse | ATCCGACAAGTTTGTACAAAAAAGCAGGCTTT**A**TGATCTGCTGTGACAAAGTCTTGTCG  CTATACCACTTTGTACAAGAAAGCTGGGTT**G**AAGGAGTGTGATCGAGAACGGCTGTT | pENTR118 |
| *ct135* | Forward  Reverse | GCCGAACAAGTTTGTACAAAAAAGCAGGCTTT**A**TGGTAAGCTTCGATTTAAATGATCCA  CGATACCACTTTGTACAAGAAAGCTGGGTT**C**TCTATACGCGCATCTAAAGGACTTGC | pENTR135 |
| *ct224* | Forward  Reverse | AATTCACAAGTTTGTACAAAAAAGCAGGCTTT**A**TGAGTTTTGTTGGAGAT  CATATCCACTTTGTACAAGAAAGCTGGGTT**A**TCATTGGGAAAAATTGAGT | pENTR224 |
| *incC* | Forward  Reverse | GCCGCACAAGTTTGTACAAAAAAGCAGGCTTT**A**TGACGTACTCTATATCCGATATAGCA  GCGGACCACTTTGTACAAGAAAGCTGGGTT**G**CTTACATATAAAGTTTGAGGATCAGA | pENTR233 |
