# Supplementary material for: Characterization of interactions between inclusion membrane proteins from Chlamydia trachomatis
Source: Front Cell Infect Microbiol. 2015 Feb 11;5:13. doi: 10.3389/fcimb.2015.00013 (PMC4324299; doi:10.3389/fcimb.2015.00013)
Supplement: Supplementary file 5 [file Table5.DOCX]

**Table S5: BACTH analysis of hetero-oligomerization of the Inc proteins**

The interactions identified with the BACTH system are listed in this table. All Inc proteins were fused to the C-terminus of T25 or T18. An interaction corresponds to a β-galactosidase activity of the DHT1 bacteria at least 5 times superior to the negative control (<10 RU). The homotypic interactions are in bold.

| Bait Protein T25 fusion | | MW^1^  (Da) | Developmental expression^2^ | | Prey protein  T18 fusion | |  | | β-galactosidase activity values (RU)^3^ |  |
| --- | --- | --- | --- | --- | --- | --- | --- | --- | --- | --- |
| Ct005 | 39537 | | | early | | **Ct005**  IncA  IncF | |  | **132 (11)**  119 (14)  59 (38) | |
| Ct058 | 40072 | | | mid | | IncD | |  | 192 (39) | |
| Ct115 incD | 14912 | | | early | | **IncD**  Ct058  Ct222 | |  | **237 (41)**  178 (86)  144 (27) | |
| Ct117 incF | 10420 | | | early | | **IncF**  IncA  IncC  IncD  IncG  Ct005  Ct058  Ct249  Ct850 | |  | **461 (61)**  197 (53)  126 (43)  319 (35)  152 (16)  113 (39)  105 (29)  277 (65)  603 (97) | |
| Ct118 incG | 17389 | | | early | | IncD | |  | 179 (29) | |
| Ct119 incA | 30313 | | | mid | | **IncA**  IncC  IncD  IncF  Ct005 | |  | **218 (43)**  107 (31)  156 (25)  157 (37)  138 (33) | |
| Ct222 | 13915 | | | mid | | **Ct222**  Ct850  Ct223  Ct224  IncD | |  | **257 (21)**  169 (77)  116 (22)  136 (10)  266 (59) | |
| Ct223 | 29475 | | | mid | | **Ct223** | |  | **176 (46)** | |
| Ct225 | 13264 | | | mid | | **Ct225** | |  | **103 (14)** | |
| Ct229 | 23422 | | | early | | Ct222  Ct223  IncD | |  | 145 (26)  156 (10)  354 (86) | |
| Ct233 incC | 18418 | | | early | | **IncC**  IncA | |  | **303 (34)**  74 (18) | |
| Ct249 | 12210 | | | early | | **Ct249**  IncF | |  | **306 (80)**  347 (58) | |
| Ct813 | 29570 | | | mid | | **Ct813** | |  | **198 (42)** | |
| Ct850 | 45822 | | | mid | | Ct222  IncF | |  | 92 (18)  619 (52) | |

^1^ Deduced from protein sequence

^2^ Developmental expression pattern as deduced from qPCR analysis

^3^ The β-galactosidase activity values are in Relative Unit
